# Reply to Yap, C.K.; Ong, M.C. Comment on “Peycheva et al. Trace Elements and Omega-3 Fatty Acids of Wild and Farmed Mussels (*Mytilus galloprovincialis*) Consumed in Bulgaria: Human Health Risks. *Int. J. Environ. Res. Public Health* 2021, *18*, 10023”

**DOI:** 10.3390/ijerph20146411

**Published:** 2023-07-20

**Authors:** Katya Peycheva, Nicola Cicero

**Affiliations:** 1 Department of Chemistry, Medical University of Varna, 9002 Varna, Bulgaria; peytcheva@hotmail.com; 2 Department of Biomedical and Dental Sciences and Morphofunctional Imaging, University of Messina, 98168 Messina, Italy; 3 Science4Life, Spin off Company, University of Messina, 98168 Messina, Italy

The authors would like to thank Yap and Ong [1] for their thorough reading and strong interest in our paper entitled “Trace Elements and Omega-3 Fatty Acids of Wild and Farmed Mussels (*Mytilus galloprovincialis*) Consumed in Bulgaria: Human Health Risks”. *Int. J. Environ. Res. Public Health* 2021, *18*, 10023. We highly appreciate their insightful and helpful comments which will improve the understanding and potential impact of our paper.

Here, we will allow ourselves to add some notes and comments. Several authors in their studies in highly cited journals cite the oral carcinogenic potency slope for Ni based on the EPA Region III Risk-Based Concentration Table as 1.7 μg/g/day [2,3]. In order to assess the target cancer risk (TR), we had accepted and applied it in the present work. We are willing to introduce an additional footnote in Table 5, subject to the approval of the the Academic Editor(s), and update the original publication.

Concerning the TR_Ni,,_ we would like to point out that the values were in the range of 1.5 × 10^−6^ to 5.1 × 10^−6^. The level of total cancer risk that is of concern is a matter of personal, community, and regulatory judgment; risks above 1 × 10^−4^ may be sufficiently large that some sort of remediation is desirable. Excess cancer risks that range between 1 × 10^−6^ and 1 × 10^−4^ are generally considered to be acceptable, although this is evaluated on a case-by-case basis and the EPA may determine that risks lower than 10^−4^ are not sufficiently protective and warrant remedial action [4,5].

Keeping in mind all the above statements, the authors state that the scientific conclusions are unaffected.

Further, as Yap and Ong [1] commented, there is an urgent need to establish target risk limits for elemental Ni in food via oral intake in the near future and we would be more than grateful to have the opportunity to participate in such types of debates and support further research programs in the field.
